# Chicago Dental Society

**Published:** 1891-05

**Authors:** Thos. L. Gilmer

**Affiliations:** Cor. Sec'y.


CHICAGO, April 9th, 1891.

*Editor of the American Journal of Dental Science:*

At the annual meeting of the Chicago Dental Society, held Tuesday evening, April 7th, 1801, the following officers were elected for the ensuing year:

President, D. M. Cattell; 1st Vice-President, J. W. Wassall; 2nd Vice-President, E. M. S. Fernandez; Recording Secretary, L. L. Davis; Corresponding Secretary, T. L. Gilmer; Treasurer, E. D. Swain; Librarian, A. W. Harlan; Executive Committee, J. A. Quinn, G. H. Cushing, E. Noyes; Board of Censors, B. S. Palmer, G. J. Dennis, R. M. C. Paine.

THOS. L. GILMER, *Cor. Sec'y.*

